# Single-incision laparoscopic myomectomy

**DOI:** 10.4103/0972-9941.72391

**Published:** 2011

**Authors:** Tiffany R Jackson, Jon I Einarsson

**Affiliations:** Department of Obstetrics and Gynecology, Minimally Invasive Gynecologic Surgery, Brigham and Women’s Hospital, 75 Francis Street, Boston, MA

**Keywords:** Laparoscopic myomectomy, single-incision, single port

## Abstract

Laparoscopic myomectomy is a minimally invasive surgical option for the treatment of uterine leiomyomas. Single-incision laparoscopy is a relatively new concept that has potential in gynaecological surgery although the technical challenges of single-incision access have limited the widespread use of the technique. The use of intracorporeal suturing is a significant component of the learning curve for laparoscopic myomectomy and presents an even greater challenge with single-incision laparoscopic myomectomy. This article describes a surgeon’s approach to single-incision laparoscopic myomectomy.

## INTRODUCTION

Uterine leiomyomas are found in as many as 70% of women and can cause symptoms such as pelvic pressure and abnormal uterine bleeding.[1] Laparoscopic myomectomy is a safe minimally invasive treatment option for uterine leiomyomas.[2] The benefits of laparoscopic myomectomy over laparotomy include lower postoperative analgesic use and a faster postoperative recovery.[3]

Laparoscopic single-incision surgery offers patients the suggested benefits of improved cosmetic appearance and the theoretical benefits of decreased pain and postoperative morbidity when compared with conventional multiple port access laparoscopy. Although single-incision surgery has been employed for over 40 years in minor gynaecological procedures, the widespread use of single-incision surgery for major gynaecological procedures has been limited to gynaecologists with advanced laparoscopic skills. Single-incision laparoscopic myomectomy is procedure anticipated to likely be in low volume during the infancy of single-incision gynaecological surgery due to the surgical expertise required.[4] Single-incision laparoscopic myomectomy, however, is a feasible and safe surgical technique.[5] This article presents an example of an approach to single-incision laparoscopic myomectomy.

## PREOPERATIVE PREPARATION

Preoperative patient counseling must include that multiple laparoscopic ports may be required to safely and successfully perform the procedure. The patient may be aware of suggested benefits of improved cosmetic outcome and decreased postoperative pain; however, they should also be aware that the potential benefits have not been evaluated thoroughly with prospective trials.

Preoperative preparation also includes deciding upon the operative technique and also the specialized equipment to be used to help overcome some of the technical challenges unique to single-incision surgery. The difficulties associated with single-incision surgery include limited working space outside of the abdomen for the surgeon and the assistant resulting in hand collisions at or above the umbilicus. Additionally, within the abdomen there is limited ability for triangulation or confinement to a single-axis workspace for instruments. Specialized equipment for single-incision procedures can be used to help overcome these technical challenges including the use of specialized access ports, articulating instruments, a flexible laparoscope or a 30° laparoscope, and instruments of varying lengths. Although these specialized instruments can make single-port procedures easier to perform they can be fairly complex to operate and are generally associated with increased costs compared with the instrumentation used with conventional laparoscopy.

A difficulty for surgeons with laparoscopic myomectomies is the requirement for significant use of intracorporeal suturing for uterine closure. The single-incision technique adds an extra dimension of difficulty to this step. The myometrium can be closed with barbed suture which has been shown to be feasible and faster for myomectomies.[6] The use of bidirectional barbed suture is safe for uterine closure and facilitates closure through preventing back sliding of the suture and eliminating the need for knot tying.[7] Specific use of barbed suture during a single-incision laparoscopic myomectomy is advantageous as intracorporeal knot tying is more challenging with a single-incision laparoscopic approach rather than with multiport access laparoscopy. The barrier of intracorporeal suturing has been facilitated for some surgeons by the use of Da Vinci (Intuitive Surgical, Sunnyvale, CA) robot-assisted laparoscopic myomectomies. Although single-incision robot-assisted laparoscopic myomectomies have not been described, the use of robot assistance for other single-incision gynaecological laparoscopic surgery is feasible.[8]

The single-incision laparoscopic myomectomy described in the article will modify the surgeon’s technique from a conventional multi-port laparoscopic myomectomy only by the difference in port placement, the use of extra long bronchoscope for optics and manual myoma morcellation.

## POSITIONING PATIENT AND PORTS

The patient is placed in dorsal low lithotomy position and a Vcare (ConMed Corp., Utica, NY) uterine manipulator and a Foley catheter are inserted. Specifically manufactured access port devices used in gynaecological surgery include, but are not limited to: SILSport (Covidien, Norwalk, CT), TriPort and Quadport (Advanced Surgical Concepts), Gelpoint(Applied Medical, Rancho Santa Margarita, CA) and AirSeal (Surgiquest, Orange, CT). The author will use a “Single-port access technique” developed by Curcillo and King, which permits greater degrees of triangulation.[9]

**Figure 1:**
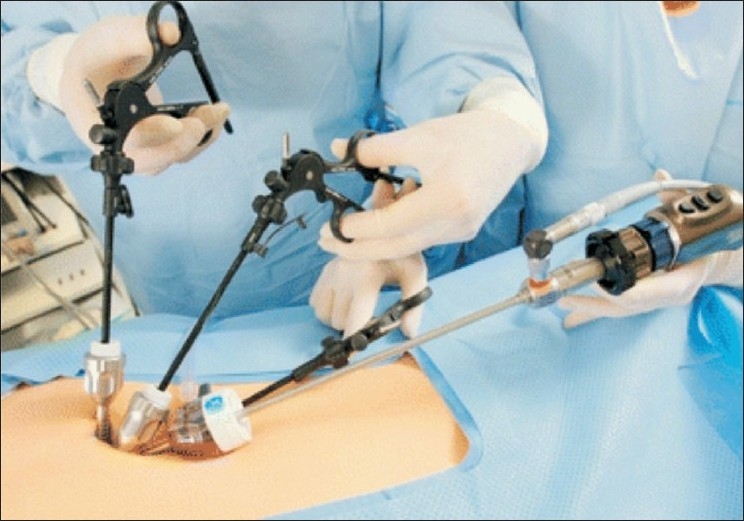
External Trocar Arrangement using the “Single Port Access (SPA) Technique” developed by Curcillo and King. Reprinted with kind permission from Springer Science Business Media. Podolsky ER, Curcillo PG 2^nd^. Single-port access (SPA) surgery-a 24-month experience. J Gastrointest Surg 2010;14:759-67.

A 2-cm vertical skin incision is made at the base of the umbilicus and a Veres needle is inserted into the peritoneal cavity. The abdomen is insufflated with carbon dioxide and the incision is extended beneath the skin by dissecting the subcutaneous tissue from the underlying fascia bilaterally using the Metzenbaum scissors. This creates flaps of skin and subcutaneous tissue and the linear incision now becomes circular. This allows for placement of three trocars within the single-skin incision, however, with each trocar placed through a separate fascial incision. A 5-mm optical trocar is placed under direct visualization and the two low-profile 5-mm trocars are inserted slightly caudal and lateral bilaterally to the initial trocar while using a 30° scope to visualize entry [Figure 1]. A bronchoscope, extra long length, with a 30° angle of view, is used. Using instruments of varying length moves the camera head away from the two operating instruments, again allowing for more freedom of movement outside the abdomen.

## OPERATIVE STEPS

**Figure 2:**
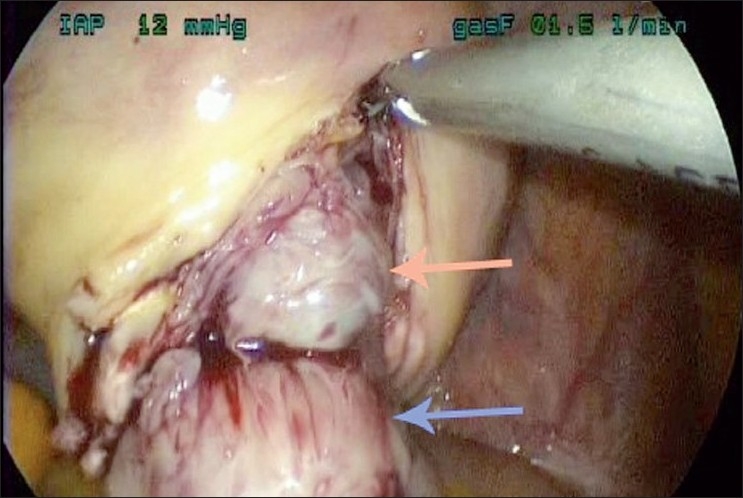
Fibroid removal. Pink arrow indicates endometrium, blue arrow indicates fibroid

**Figure 3:**
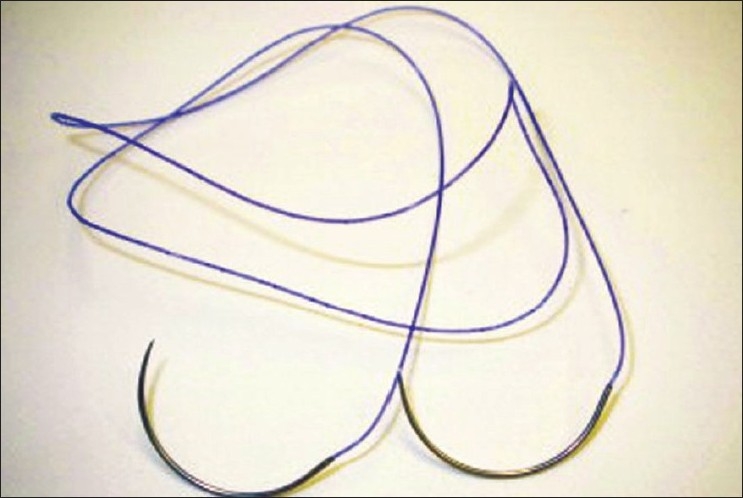
Quill Barbed Suture.

After the ports are inserted, the subserosa of the largest myomas is injected with dilute vasopressin, 20 units in 40ml of saline solution, using only 10 ml with each injection. A vertical incision is made over the myoma using the Harmonic Ace (Ethicon, Cincinnati, OH) and the myoma is grasped with a single-toothed tenaculum. Traction is placed on the tenaculum and the myoma is released from the uterus using the Harmonic Ace and blunt dissection [Figure 2]. The myoma is placed in the posterior cul-de-sac. Attention is turned to closing the myometrium. The 14×14-cm 0-polydioxanone bidirectional barbed Quill suture (Angiotech, Vancouver, BC) is placed through a 5-mm port by straightening the needles on each end [Figure 3]. Once inside the abdomen, the needles are curved back again using a grasper and needle driver. One needle is placed in the peritoneum of the abdominal wall while the other needle is used for suturing.

**Figure 4:**
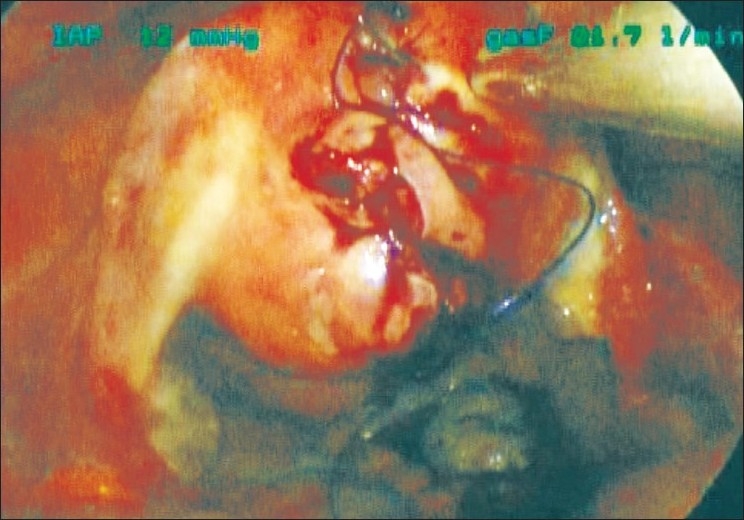
Suturing using the Quill suture

**Figure 5:**
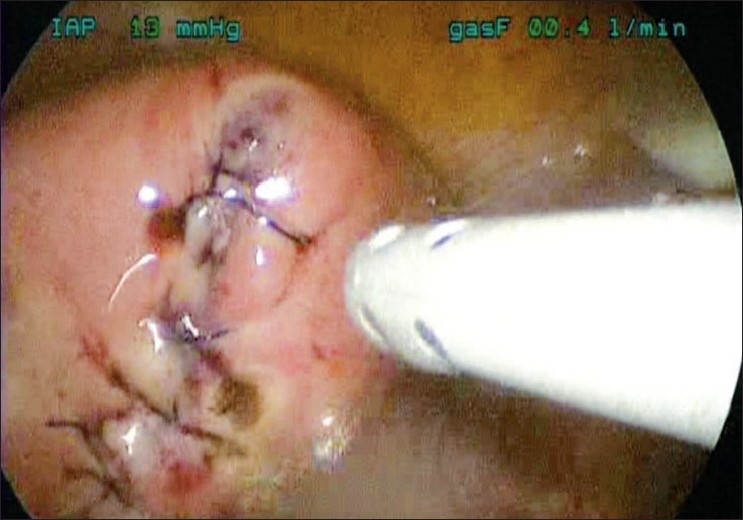
Uterus at the completion of the hysterotomy closure

A three-layer closure of the uterus is then performed [Figures 4 and 5]. The deepest layer of myometrium is closed with the first needle; the needle is cut off, straightened and removed from the abdomen. A more superficial layer is closed with the second needle and likewise the needle is cut off, straightened and removed from the abdomen. Another barbed suture is introduced into the abdomen and the final layer of the hysterotomy is closed starting in the middle of the hysterotomy with the first needle drawing the suture through all the way to the midpoint of its length and suturing toward the inferior aspect of the hysterotomy. The second needle is used to suture towards the superior aspect of the hysterotomy. The two needles are cut off, straightened and removed through the trocars. No knot tying is required. Gynecare Interceed (Ethicon, West Somerville, NJ) adhesion barrier is cut in half and introduced through the trocar and placed over the hysterotomy. The myoma is brought up to the incision using the tenaculum. The three 5-mm ports are removed and the three fascial incisions are connected with the knife as the myoma is pulled further into the incision. The myoma is carefully morcellated at the level of the incision using a knife. The fascia is closed with 0-vicryl suture. The skin is closed with interrupted 3-0 monocryl suture. Ten millilitres of 0.5% bupivacaine is injected into the incision site and Dermabond (Ethicon, West Somerville, NJ) adhesive is applied over the incision. The uterine manipulator is removed and a diagnostic hysteroscopy is performed to confirm a normal endometrial cavity. The Foley catheter is also removed at the conclusion of the procedure. A single-incision laparoscopic myomectomy as described is completed by this surgeon in approximately 1–2 hours.

## POSTOPERATIVE CARE

The patient is awakened from anaesthesia and observed in the recovery room for 3–4 hours. She is observed until she can tolerate oral pain medication, void spontaneously and has adequate pain control. The patient is then discharged home and returns to the office in 2 weeks for postoperative follow-up.

## CONCLUSION

Single-incision laparoscopic myomectomy is a feasible procedure. The techniques of limited electrosurgery and multilayered closure of the myometrium developed for abdominal myomectomy should still be applied in the case of single-incision laparoscopic myomectomy.[10] The most challenging aspect of the procedure is the extensive intracorporeal suturing necessary which is achieved by this surgeon through the use of barbed suture which also eliminates the need for knot tying. Prospective studies of the safety and the long-term outcomes of single-incision laparoscopic myomectomy are needed. The costs of special equipment and possible increased surgical time involved in accomplishing the single-incision technique will also need to be assessed. As the reported cosmetic benefit of single-incision surgery is attained it is possible that patients will begin to request such procedures and that further innovation will occur to simplify this procedure for more surgeons.
